# The immunomodulatory activities of licorice polysaccharides (*Glycyrrhiza uralensis* Fisch.) in CT 26 tumor-bearing mice

**DOI:** 10.1186/s12906-017-2030-7

**Published:** 2017-12-15

**Authors:** Peter Amwoga Ayeka, YuHong Bian, Peter Mwitari Githaiga, Ying Zhao

**Affiliations:** 1 International College of Chinese Materia Medica, Tianjin University of Traditional Chinese Medicine, 88 Yuquan Road, 312 Anshan Western Road, Nankai District, Tianjin, 300193 People’s Republic of China; 2 Department of Biological Sciences, Faculty of Science, Egerton University, PO BOX 536-20115, Egerton, Kenya; 3 Center for Traditional Medicine and Drug Research, Kenya Medical Research Institute, P.O. Box 54840-00200, Nairobi, Kenya

**Keywords:** Licorice, Immunomodulation, Anticancer, Polysaccharides, Tumor

## Abstract

**Background:**

The increasing use of complementary and alternative medicine (CAM) has kindled the need for scientific evaluation of the mechanism of action of CAMs. Although, licorice, a common ingredient in many Traditional Chinese medicine (TCM) has attracted great attention for its antitumor and immunomodulatory activities, the mechanism of action of its polysaccharides is still unclear. Here we report the immunomodulatory activity of licorice polysaccharides in vivo.

**Methods:**

The differential anticancer activities of licorice polysaccharides by tumorigenesis and immunomodulation was evaluated in vivo. Six weeks old, 120 CT-26 tumor bearing BALB/c mice, weighing 20 ± 2 g were used. They were randomly divided into six groups, three groups receiving high molecular weight (fraction A), low molecular weight (fraction B) polysaccharides and crude extract (fraction C); positive, negative and normal groups receiving cytoxin, saline and normal diet respectively. Weight of mice and tumors was determined and tumorigenicity assay calculated to determine the anticancer effects. Immunomodulatory potential was determined by immune organ indices, immune cell population and serum cytokine levels using immune organ weight and index, flow cytometry and cytokine/chemokine bead panel kit respectively.

**Results:**

Licorice polysaccharides exhibited immunomodulatory activities in CT 26 tumor bearing BALB/c mice. The polysaccharides significantly suppressed tumor growth and increased immune organ index. Furthermore, the immunomodulatory effect was evident with activation of CD4^+^ and CD8^+^ immune cells population. The polysaccharides also affected the production of various cytokines, by increasing IL 2, IL 6, IL 7 levels and a decreasing TNFα levels.

**Conclusion:**

In summary, licorice polysaccharide especially of low molecular weight exhibit anticancer and immunomodulatory activities by suppressing tumor growth and improving general health of mice. They also augment the thymus/spleen index and population of T lymphocytes. Furthermore, the polysaccharides enhance the levels of serum antitumor cytokines, IL 2, IL 6 and IL 7 while decreasing pro-tumor cytokine TNFα.

## Background

According to global cancer burden, cancer is one of the leading causes of death worldwide, and in 2013, it was rated the second after cardiovascular diseases. With an estimated 70% increase in new cases and death, it is estimated that it will rise from 12.7 million to 21.4 million new cases and 13 million cancer death by the year 2030 [1–3]. Due to the burden caused by cancer and side effects associated with target therapies, researchers have focused on better strategies to treat and manage cancer. Currently, researchers have focused on cancer immunotherapy, which pose mild and manageable effects than side effects associated with conventional therapies [4, 5]. The immune therapies are either applied individually or combined with or after chemotherapy, radiotherapy and surgery [4, 6]. Immunomodulation in cancer therapy involves immune cells that are actively involved as antigen presenting cells (APCs), activation of T lymphocytes, cytokine production, suppression of inflammation and direct cytotoxicity to cancer cells [5, 7]. Therefore, compounds with immunomodulatory activity are of great importance in cancer immunotherapy.

Plant based extracts including those from fungi, algae and lichens have elicited great passion among researchers due to their immunomodulatory and anticancer potential. Of importance, is Licorice (*Glycyrrhiza uralensis* Fisch*.*), a promising immunomodulatory and anticancer herb and a common ingredient in major prescriptions in Chinese medicine. Its pharmacological important compounds include triterpenes, saponins, flavonoids and phenolic compounds among others [8, 9]. In Our previous studies, for instance, licorice polysaccharides exhibited anticancer and immunomodulatory potential in vitro. The polysaccharides up-regulate expression of antitumor cytokine, IL-7, enhance secretion of IL-7 by mouse intestinal epithelial cells (IEC) and stimulates T lymphocytes [10]. Isoliquiritigenin, glycyrrhizin and glabridin, licorice compounds, show anti-proliferative and apoptotic properties by acting directly on cancer cells in vitro [11, 12]. Immunomodulation potential of licorice compounds is also exhibited by 18β-glycyrrhetinic acid which up-regulates T cell proliferation, increase blood leukocyte count and spleen weight in mice [13–15]. These compounds have also been found to enhance apoptosis in cancer cells through upregulating c-myc and c-jun oncogenes and increasing activity of protein kinase C [16]. Licorice polyphenols, liquiritin, isoliquiritin and isoliquiritigenin, have demonstrated varying effects that range from cytotoxicity, apoptosis via caspase and Bcl-2 cascades, augmenting expression of Bax and Bid proteins and downregulating Bcl-2, which are initiated through caspase pathway [17]. In 4 T1 tumor model, licorice extracts inhibit metastasis of cancer cells and delay tumor progression. They also reduce tumor growth, inhibit tumor angiogenesis, inflammation and lymphangiogenesis [18, 19], which is an essential attribute for a pharmacological cancer drug. In addition, concurrent use of licorice compounds and chemotherapeutic treatment of cisplastin show increased anticancer activity and hepatoprotective potential in mice and rats [20, 21]. Furthermore, licorice extracts have the ability to downregulate expression of pro-inflammatory cytokines, tumor necrotic factor alpha (TNFα), interleukin 1 (IL-1) and interleukin 6 (IL-6) [22]. Despite this activities, immunomodulatory mechanism of action of licorice polysaccharides in vivo is still unclear. In this study, we demonstrate the immunomodulatory activities of licorice polysaccharides in CT26 tumor bearing mice.

## Methods

### Materials and animals

All chemical reagents were purchased from Sigma (Sigma-Aldrich Inc., St. Louis, MO, USA unless otherwise stated). Licorice (*Glycyrrhiza uralensis)* extracts were extracted and provided by the TUTCM herbal pharmaceutical company, Tianjin, China. The polysaccharides were fraction A of over 100 kDa was 81.4% (High molecular weight), fraction B, of 75 kDa was 45.4% and 54.6% polysaccharide fractions was under 10 kDa (Low molecular weight) and fraction C, 34.5% polysaccharide fractions was 290 kDa and 14 kDa was 30.3%, total crude extract. BALB/c CT-26 tumor bearing mice (Institute of animal research in Beijing, China).

### Preparation of licorice polysaccharides

Briefly, dried roots of Licorice (*Glycyrrhiza uralensis)* (1000 g) was extracted by boiling with 95% ethanol (*v*/v) for 2 h (h). The mixture was filtered and air dried and extracted with 10% v/v boiling water twice, 2 h each time followed by centrifugation. The aqueous extract was concentrated to 1.5 L under vacuum. Subsequently, 95% ethanol was added to the aqueous extract up to 80% to precipitate the polysaccharides and kept at 4 °C overnight. After centrifugation, the crude licorice polysaccharides (LP) were dried by forced air at 50 °C and weighed. The crude LPs were completely dissolved in aqueous solution at 5% followed by ultraphonic for 1 h (80HZ, 30 °C) and stirred for 4 h at room temperature. The crude LPs solution was centrifuged at 6000 rpm/min for 10 min. The supernatant was enriched and divided into 3 fractions (GP-A, GP-B and GP-C) for alcohol precipitation by addition of 95% ethanol with stirring homogeneously for 2 h. The final alcohol concentration was different for the 3 fractions (LP-A: 57%; LP-B: 57~82%; LP-C: 82%) and kept at 4 °C overnight. Polysaccharide pellets were obtained by suction filtration and repeatedly washed sequentially with anhydrous ethanol, acetone and diethyl ether respectively. The proteins in the extract were removed using the Savage reagent. The LPs solutions of 3 fractions were dialyzed against distilled water for 36 h and freeze dried for further experiments.

### Antitumor activity of *G.uralensis* polysaccharides in vivo

Use of animals in this study was approved by Tianjin University of Traditional Chinese Medicine Animal Care and Use Committee. One hundred and twenty (120) CT-26 tumor bearing BALB/c mice, 6 weeks old weighing on average 20 ± 2 g were used. The animals were housed in an animal facility well light by natural light, well ventilated and temperature maintained at 22 ± 2 °C. They were kept in groups of ten mice per cage and received normal mice feed and water ad libitum. Animal care and handling was performed in accordance with the guidelines for animal experiments approved by the animal care and use authorization committee of TUTCM. After three (3) days of acclimatization, mice were randomly sorted into six groups of twenty mice for further experimentation. Three fractions of licorice extracts (Fraction A, B and C) were orally administered to mice, 500 mg/kg once daily for 14 days. Cyclophosphamide (Cytoxin) was administered intraperiteonal at 50 mg/kg of mice for 10 days as positive control, the vehicle was given to negative control and normal mice received normal diet.

### Effect of polysaccharides on health of mice

The weight of the animals were taken at 0, 5, 10 and 15th day. After the treatment period, mice were sacrificed and tumor weight in each group recorded. The net weight of mice was determined by subtracting tumor weight from final body weight of mice. The changes in body weight of mice in different groups were also evaluated. General health of mice was closely monitored by observing activeness, grooming and feeding behaviour.

### Euthanasia of mice

After taking weight and observing general health, mice were euthanised and sacrificed for successive assay. Briefly, intraperitoneal injection (IP) of sodium pentobarbital (250 mg/kg) was administered to the mice [23]. This was followed by excision of tumors and immune organs.

### Tumorigenicity assay

Tumor growth was monitored for 14 days after which the animals were sacrificed. The tumor suppression ratio was determined by the following formula:

Tumor suppression (%) = [(A-B)/A] × 100. Where A, is average tumor weight of the negative control, B, is the weight of the treatment group.

### Relative immune organs weight

After treatment period, mice were sacrificed, and the spleen and thymus weight and indices were measured and calculated. The thymus and spleen indices were expressed as thymus or spleen weight relative to body weight.

### Immune cells population

One hundred and twenty CT-26 tumor BALB/c mice were equally randomised into six groups. Each group was treated with the corresponding treatment according to the experimental design for 14 consecutive days. To determine the effect of the polysaccharides on immune cell population, blood was drawn by retro-orbital puncture after treatment period. T lymphocytes subsets (CD4^+^ and CD8^+^) from peripheral blood were measured and analysed on FACS Calibur. Briefly, whole blood was drawn into EDTA pre-coated 1.5 ml tubes by rectal-orbital puncture and mixed, 100 μl added to the bottom of a well labelled tube and appropriate primary antibody added to each tube; to 100 μl sample was added 0.25 μg of FITC Hamster Anti-Mouse CD3e, 0.25 μg of APC Rat Anti-Mouse CD4 and 1.0 μg of PE Rat Anti-MouseCD8a (Biolegend Co. Ltd). The tubes were capped, vortexed and incubated in the dark for 25 min at room temperature, followed by addition of 1.5 ml lysing solution vortexed, incubated at room temperature in the dark for 10-15 min and centrifuged for 5 min at 200 x g. The supernatant was aspirated; cells fixed by re-suspending in 2% paraformaldehyde buffer for 30 min at room temperature and washed. The cells were finally suspended in 500 μl wash buffer and stored for 24 h at 4 °C in the dark. A negative control of 50 μl of cells plus 100 μl PBS following the same procedure, was used. The samples were then analysed on FACS Calibur (Merck-Millipore, Bio-Rad Beijing) with InCyte software version 2.2.2.

### Serum cytokine levels

To determine the effect of licorice polysaccharides on cytokine production, whole blood was drawn from mice after last day of treatment by rectal-orbital puncture, centrifuged at 4 °C for 3000×g for 15 min, serum obtained and frozen at -20 °C until use. The cytokine levels in serum were measured by mouse cytokine/chemokine magnetic bead panel kit 96-Well Plate Assay (Milliplex MAP) Shanghai, China. The assay was run on Luminex 200 MAGPIX® with xPONENT software and analyzed for Median Fluorescent Intensity (MFI) data using a 5-parameter logistic or spline curve-fitting method for calculating cytokine/chemokines concentrations in samples.

### Statistical analysis

Data were expressed as Mean ± SD. Normality of data was checked by Shapiro-wilk test and data was normally distributed. Statistical comparison between groups was carried out using one-way ANOVA followed by separation of means by Bonferroni. This was performed using SPSS Version 20.0. Curve–fitting was done using GraphPad Prism version 5 package. Values were considered statistically significant at *P-value* < 0.05.

## Results

### Mice weight and health

After emergence of tumors and commencement of treatment, there was remarkable change in the general health of the treated mice. This was observed through state of grooming, activity and feeding behaviour. As shown in Table 1, tumor mice treated with licorice polysaccharides, fraction B, had an increase in weight, 25.03 ± 4.3 g and a low tumor weight of 5.96 ± 0.8 g. It had minimal change in weight and physical appearance. It’s activity, grooming, eating and fur appearance did not change compared to saline group, which were emaciated and dull. The other treatment groups including the cytoxin group, had a reduction in total weight with saline group having highest weight loss. Notably, the results indicated that, although there was initial weight loss in all groups, there was a clear change in this trend, with the treated groups gaining weight. Fraction B treated group, weight (19.07 g), had the highest final weight compared to saline group, although the weight was lower than that of normal mice (20.56 g).

**Table 1:** Effect of licorice polysaccharides on weight of mice and tumor suppression

Treatment Group	Mice with tumor(g)	Tumor weight(g)	Mice without tumor(g)	Tumor suppression (%)
Normal	20.56 ± 1.3	0	20.56 ± 1.3	–
Saline	23.48 ± 2.7	7.41 ± 0.4	16.07 ± 2.3*	–
Cytoxin	23.51 ± 3.2	5.05 ± 1.1^#^	18.46 ± 2.1	31.85
Fraction A	25.60 ± 2.4*	7.31 ± 0.9	18.32 ± 1.5	1.35
Fraction B	25.03 ± 4.3*	5.96 ± 0.8^#^	19.07 ± 3.5	19.57
Fraction C	24.74 ± 2.5	6.59 ± 1.0	18.19 ± 1.5	11.07

Data represented as mean ± SD, *n* = 10. *p < 0.05 versus Normal group; ^#^ *p* < 0.05, versus Saline group

### In vivo tumor suppression

To investigate in vivo antitumor activity of licorice polysaccharides, a successful model of CT-26 tumor bearing BALB/c mice were treated orally with various concentrations for 14 days. The three licorice polysaccharide fractions showed varied tumor suppression rates. From the results in Table 1 above, Fraction B, had a significantly higher tumor suppression rate among all groups albeit, lower than cytoxin treated group, that is, 19.57% and 31.85% respectively while fractions A had the lowest tumor suppression rate.

### Relative immune organ weight and indices

Immunomodulatory activity of licorice polysaccharides was evaluated by determining their effect on immune organs, thymus and spleen. There was a significant increase in spleen and thymus weight (*p* ≤ 0.05) in fraction B treated group compared to saline group. There was also a significant change in spleen and thymus indices. Fraction B had a significant high thymus index of 0.637 ± 0.14 compared to saline treated group. Although, there was no significant difference in spleen index between all the groups, the highest spleen index was observed in Fraction B treated group (Table 2).

**Table 2:** Effect of Licorice polysaccharide on immune organs index

Index	Normal	Saline	Cytoxin	Fraction A	Fraction B	Fraction C
Spleen mg	80 ± .002	220 ± .015	160 ± .028	200 ± .010	250 ± .029*	190 ± .014
Thymusmg	26.63 ± 2.10	9.87 ± .66	10.2 ± .71	13.56 ± .54	15.42 ± .44*	13.67 ± .53
Spleen index	.004 ± .00	.009 ± .00	.007 ± .00	.008 ± .00	.010 ± .00	.008 ± .00
Thymus index	1.29 ± .26	.423 ± .08	.433 ± .06	.532 ± .06	.637 ± .14*	.555 ± .07

Data expressed as mean ± SD.*p < 0.05 versus saline group. *n* = 10

### Immune cell population

**Fig. 1:**
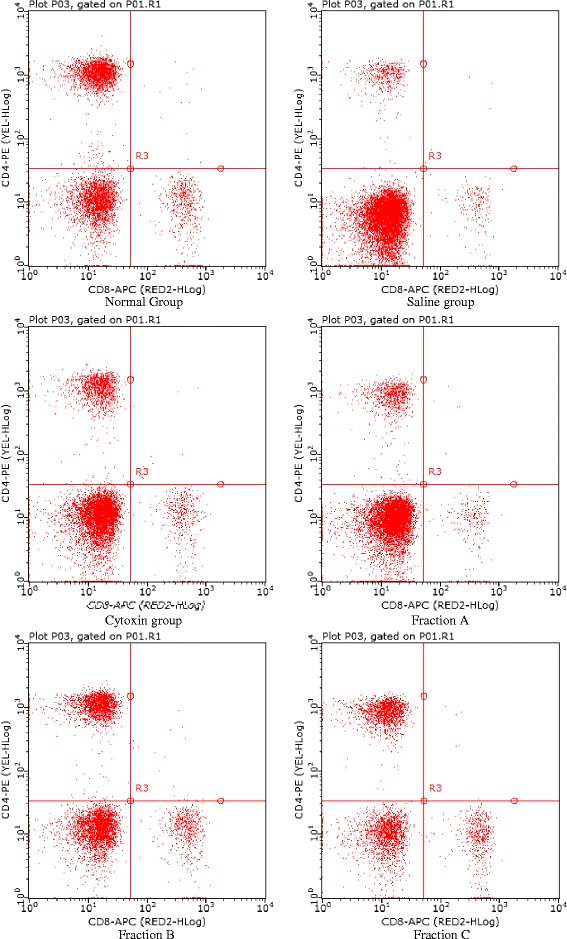
Dot plots for representative treatment groups of Normal, Saline, Fraction A, Fraction B and Fraction C after treatment period. To determine immune activity of licorice polysaccharides on proliferation T lymphocytes through stimulation, flow cytometry assay was carried out. Whole blood was collected by retro-orbital puncture and stained with respective markers, CD3, CD4 and CD8. Normal group had a high percentage of CD4 and low CD8; Cytoxin group, high CD4 and low CD8; Fraction A, high CD4 and low CD8; Fraction B, high CD4 and low CD8 and Fraction C, low CD4 and high CD8 cells compared to Saline treated group

The immunomodulatory effect via activation of T lymphocytes by licorice polysaccharides in CT-26 BALB/c tumor model mice was analysed by flow cytometry. After treatment period, T lymphocytes were analysed from peripheral blood and the relative levels of CD4^+^ and CD8^+^ T lymphocytes subsets determined. From the results (Table 3 and Fig. 1), there was a significant increase in the percentage number of CD4^+^ T lymphocytes subset in all the groups except Fraction C. Fraction B treated group showed the highest percentage of 78.70 ± 3.4%, saline group 75.74 ± 4.7% and normal group 79.82 ± .1.1%, with Fraction C having the lowest count of 71.20 ± 0.9%. The percentage population of CD8^+^ subset of T lymphocytes was significantly reduced in all groups except Fraction C which showed a significant increase.

**Table 3:** Effect of Licorice polysaccharides on T lymphocyte population

TT Cells, %	Normal	Saline	Cytoxin	FractionA	Fraction B	Fraction C
CD4^+^	79.82 ± 1.1*	72.76 ± 2.3^#^	75.74 ± 4.7^#^	77.61 ± 0.3*	78.70 ± 3.4*	71.20 ± .9^#^
CD8^+^	18.55 ± 0.9*	25.98 ± 2.4^#^	19.62 ± 4.1*	21.25 ± 0.3*	20.44 ± 3.2*	27.05 ± .6^#^

Data are expressed as mean ± SD. *p ˂ 0.05, versus Saline group; ^#^ *p < 0.05* versus Normal group, *n* = 10

### Cytokine serum levels

**Fig. 2:**
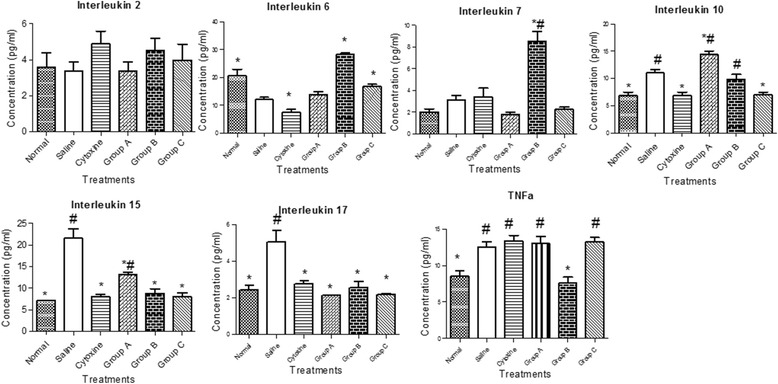
Effect of Licorice polysaccharides on cytokine serum levels. Interleukin 2,6,7,10,15, 17 and TNFα. After treatment period, mice were sacrificed and cytokine levels measured by magnetic bead panel assay. Data represented as mean ± S.D. *p˂0.05 versus the Saline group, ^#^ *p*˂0.05 versus Normal groups

We set to evaluate antitumor potential of licorice polysaccharides through immunomodulation by measuring various peripheral cytokines (Fig. 2). After treatment period, cytokines, Interleukin 2, IL-6, IL-7, IL-10, IL-15, 1 L-17 and TNFα in CT-26 tumor bearing BALB/c mice serum were analysed. The cytokine levels were measured by magnetic bead panel assay. From the results (Fig. 2), IL-2 levels increased in all treatment groups. Fraction B had the highest increase compared to other treatment groups except cytoxin treated group, even though the difference was not significant at *p* < 0.05. The serum levels of IL-6 were low in all the treatment groups except for fraction B which had significantly higher levels of 28.31 ± 1.2 pg/ml at *p* < 0.05. In comparison to saline group, all treatment groups showed a significant increase except the cytoxin group. Furthermore, all treatment groups had an increase in the levels of IL-7 except Fraction A. That is, fraction B, the highest with 8.54 ± 1.7 pg/ml and fraction A, lowest with 1.78 ± 0.5 pg/ml. The serum levels of the other cytokines including IL-10, IL-15 and 1 L-17 were lower in all the groups,except fraction A compared to saline group. When compared with the normal group, fraction B had higher levels of IL-15 and IL-17, with IL-10 being significantly high. The serum levels of TNFα were high in all the groups except fraction B and the normal group.

## Discussion

Licorice plays an important role as an ingredient in many prescriptions used in complementary and alternative medicine. In TCM, it is prescribed for many diseases while in Japan, apart from its therapeutic use, it is also used as an ingredient in confectionary. Although the therapeutic potential of licorice have been studied, extensive work on immunomodulation and anticancer potential of its polysaccharides have not been fully exploited.

This study investigated the potential of licorice polysaccharides as immune modulating agents in CT 26 tumor bearing BALB/c tumor mice models. In our previous studies, we demonstrated that licorice polysaccharides not only upregulate IL-7 expression but also inhibit proliferation of cancer cells in vitro [10]. Augmenting of IL-7 expression is of great importance in cancer immunotherapy. This is due to the fact that IL-7 is a critical cytokine in lymphopoiesis. Besides IL-7, there have been concerted efforts to use the immune system to fight cancer, which has led to study, exploitation and development of other cytokines for cancer immunotherapy. Immunomodulation in cancer immunotherapy, as an emerging therapy is associated with minimal side effects, and boosts the overall body’s immune system [24]. Therefore, agents that modulate the immune system are critical and important in the fight against cancers as opposed to conventional therapies that lead to immunosuppression.

Conventional therapies including radiotherapy, chemotherapy and surgery pose adverse secondary effects that ultimately lead to reduced quality of life, poor feeding habits, weight loss due to cachexia, recurrence of tumors and death, unlike immunotherapy that pose mild side effects and improve quality of life of patients [25–31]. In the recent past, there has been an increase in the use of complementary and alternative medicine due to its holistic treatment, less side effects and boosting of the immune system.

In this study, licorice, a common CAM, augmented the immune system in tumor mice models. The experimental mice treated with low molecular weight icorice polysaccharide, (Fraction B) had a higher body weight and small tumor size (Table 1), indicating the potential of licorice in improving quality of life of tumor hosts as well as suppressing tumor growth.. This was also evident by the behaviour of treated mice, such as, grooming, feeding and fur appearance. (Data not shown). This is contrary to common effects of conventional therapies that are associated with poor prognosis and response to drugs [32–34]. Tumor suppression has been fronted as a good indicator in cancer prognosis. In relation to tumor suppression, CAMs with anticancer compounds inhibit tumor progression [35, 36]. This can be attributed to either direct cytotoxicity to cancer cells or it could be through a cascade of events, including immunomodulation. In this study, fraction B treated group showed a significant tumor suppression rate compared to saline group (Table 1). In addition, relative spleen and thymus index are important indices for nonspecific immunity. Potent immunomodulatory compounds increase the spleen and thymus weight [36–39]. This increase is attributed to stimulation of immune cells in the spleen and thymus. The spleen is actively involved in filtration of blood and stores monocytes. Studies indicate that licorice enhances the immune status of mice by increasing blood leukocyte count and weight of the spleen [13]. In our studies, spleen and thymus weight and index were higher in fraction B treated group compared to saline group (Table 2). This phenomenon can be attributed to the immune modulating and anticancer potential of licorice polysaccharides, which is also true with other CAMs [40–42]. The spleen and thymus represent major secondary and primary lymphoid organs involved in eliciting an immune response and maturation of immune cells, and are also important in humoral and cellular immunity. The change in spleen and thymus weight and index can also be attributed to signalling events of IL-7 which is critical in stimulation, maturation and homeostasis of lymphocytes. In our previous studies, we demonstrated the ability of licorice polysaccharides to augment IL-7 expression, secretion and stimulate proliferation of T lymphocytes, thereby exhibiting immunomodulatory activity on immune organs and cells [10].

It is well documented that one of the probable mechanism of immune stimulation activity of drugs is through activation of immune cells such as CD4 and CD8 T cells. The increase in these immune cells is thought to be a good indicator of better prognosis and an active immune response to tumors and infections [43]. In the present study, licorice polysaccharide of low molecular weight (Fraction B) stimulated proliferation of CD4 and CD8 T cells compared to saline group (Table 3) with a significant increase and decrease respectively, thus, suggesting that, stimulation and activation of lymphocytes is a probable mechanism of immunomodulation by licorice polysaccharides. This findings concur with other findings that have reported the importance of CD4 T cells in mounting an effective anticancer immunity and reduced CD4 T cells enhances tumor progression [44, 45]. Moreso, CD4 T cells are known to promote antitumor activity of CD8 T cells, and the antitumor activity is augmented in presence of both CD4 and CD8 T cells [46]. The increased number of CD4 T cells in fraction B treated group, is a likely reason for the observed higher tumor suppression rate. The population of CD4 and CD8 T cells observed again can be associated with stimulation by IL-7 [10, 47].

Furthermore, studies on various anticancer CAMs have demonstrated that promotion of anti-inflammatory cytokines and/or inhibition of pro-inflammatory cytokines is one of their mechanism of action [42, 48]. For instance, IL-7 is significant in cancer immunotherapy, by promoting stimulation, activation, survival and homeostasis of immune effector cells. Due to the potential of IL-7 and other cytokines, some have been approved and are currently used clinically for immunotherapy [47, 49, 50]. In our study, there were differential immunological activities of licorice polysaccharides in CT 26 tumor bearing BALB/C mice. Fraction B upregulated the production of various cytokines such as IL-2, IL-6 and IL-7 in comparison to saline group (Fig. 2). In comparison to normal group, production of IL-10, IL-15 and IL-17 was high but lower than the saline group, while the production of pro-inflammatory cytokine, TNFα, was lower in fraction B. These cytokines, interleukin-2, IL-6, IL-15 and IL-7 are involved in activating T lymphocyte growth, survival, maturation and homeostasis. They are therefore, critical cytokines in ensuring effective antitumor immunity [10, 44, 45, 51]. The increased production following treatment with fraction B, exhibits the antitumor and immune modulating activity of these compounds. In fact, lack of IL-7 is associated with severe immunodeficiency and presence of IL-7 in blood cancer patients is an indication of better prognosis which is associated with activating immune effector cells in tumor hosts [51–53]. On the other hand, IL-10, IL-17 and TNFα, though some studies indicate that they activate cell growth, they have been associated with blocking apoptosis, inhibiting antigen presenting cells, inhibiting cytokine production, favour tumor growth and promote inflammation. TNFα, which is a pro-inflammatory cytokine, also promotes tumor development, neurovascularization and metastasis of tumors [54, 55]. Low levels of TNF-α in this study, is a good indicator of the antitumor potential of licorice polysaccharides which inhibited its production and circulation, thereby affecting and suppressing tumor growth.

## Conclusion

Current research is geared towards immunotherapy which pose mild side effects. CAMs are also gaining popularity due to their holistic approach involving modulating the immune system and as biological response modifiers (BRMs) in cancer immunotherapy. Licorice polysaccharides of low molecular weight as CAM, have the potential of immunomodulatory and anticancer compounds for cancer treatment. They are non-cytotoxic, suppress tumor growth, increase immune organ weight and index, activate immune cells and stimulate secretion of ant-inflammatory cytokines, especially IL-7, and inhibit secretion of pro-inflammatory cytokines, TNF-α. These polysaccharides therefore have a potential of use in cancer immunotherapy.

## Abbreviations

ANOVA: Analyses of variance
CAM: Complementary and Alternative Medicine
CD: Cluster of Differentiation
CT 26: Colon tumor cell line 26
IL: Interleukin
S.E.M: Standard Error of Mean
TCM: Traditional Chinese medicine
TNFα: Tumor Necrotic Factor alpha
TUTCM: Tianjin University of Traditional Chinese medicine
